# Experimental Cerebral Malaria Spreads along the Rostral Migratory Stream

**DOI:** 10.1371/journal.ppat.1005470

**Published:** 2016-03-10

**Authors:** Angelika Hoffmann, Johannes Pfeil, Julieta Alfonso, Felix T. Kurz, Felix Sahm, Sabine Heiland, Hannah Monyer, Martin Bendszus, Ann-Kristin Mueller, Xavier Helluy, Mirko Pham

**Affiliations:** 1 Department of Neuroradiology, Heidelberg University Hospital, Heidelberg, Germany; 2 Division of Experimental Radiology, Department of Neuroradiology, Heidelberg University Hospital, Heidelberg, Germany; 3 Centre for Infectious Diseases, Parasitology Unit, Heidelberg University Hospital, Heidelberg, Germany; 4 Center for Childhood and Adolescent Medicine, General Pediatrics, University Hospital, Heidelberg, Heidelberg, Germany; 5 German Centre for Infection Research (DZIF), Heidelberg, Germany; 6 Department of Clinical Neurobiology, Medical Faculty of Heidelberg University and German Cancer Research Center (DKFZ), Heidelberg, Germany; 7 Department of Neuropathology, Heidelberg University Hospital, Heidelberg, Germany; German Cancer Consortium (DKTK), Clinical Cooperation Unit Neuropathology, German Cancer Research Center (DKFZ), Heidelberg, Germany; 8 Experimental Physics V, University of Würzburg, Würzburg, Germany; 9 NeuroImaging Centre Research, Department of Neuroscience, Ruhr-University Bochum, Bochum, Germany; Michigan State University, UNITED STATES

## Abstract

It is poorly understood how progressive brain swelling in experimental cerebral malaria (ECM) evolves in space and over time, and whether mechanisms of inflammation or microvascular sequestration/obstruction dominate the underlying pathophysiology. We therefore monitored in the *Plasmodium berghei* ANKA-C57BL/6 murine ECM model, disease manifestation and progression clinically, assessed by the Rapid-Murine-Coma-and-Behavioral-Scale (RMCBS), and by high-resolution *in vivo* MRI, including sensitive assessment of early blood-brain-barrier-disruption (BBBD), brain edema and microvascular pathology. For histological correlation HE and immunohistochemical staining for microglia and neuroblasts were obtained. Our results demonstrate that BBBD and edema initiated in the olfactory bulb (OB) and spread along the rostral-migratory-stream (RMS) to the subventricular zone of the lateral ventricles, the dorsal-migratory-stream (DMS), and finally to the external capsule (EC) and brainstem (BS). Before clinical symptoms (mean RMCBS = 18.5±1) became evident, a slight, non-significant increase of quantitative T2 and ADC values was observed in OB+RMS. With clinical manifestation (mean RMCBS = 14.2±0.4), T2 and ADC values significantly increased along the OB+RMS (p = 0.049/p = 0.01). Severe ECM (mean RMCBS = 5±2.9) was defined by further spread into more posterior and deeper brain structures until reaching the BS (significant T2 elevation in DMS+EC+BS (p = 0.034)). Quantitative automated histological analyses confirmed microglial activation in areas of BBBD and edema. Activated microglia were closely associated with the RMS and neuroblasts within the RMS were severely misaligned with respect to their physiological linear migration pattern. Microvascular pathology and ischemic brain injury occurred only secondarily, after vasogenic edema formation and were both associated less with clinical severity and the temporal course of ECM. Altogether, we identified a distinct spatiotemporal pattern of microglial activation in ECM involving primarily the OB+RMS axis, a distinct pathway utilized by neuroblasts and immune cells. Our data suggest significant crosstalk between these two cell populations to be operative in deeper brain infiltration and further imply that the manifestation and progression of cerebral malaria may depend on brain areas otherwise serving neurogenesis.

## Author Summary

Brain swelling is difficult to detect ex vivo and has recently been identified as a strong predictor of death not only in experimental cerebral malaria (ECM), but also in human cerebral malaria. As whole-brain in-vivo imaging methods have been widely underutilized in this disease model, little is known about the spatiotemporal evolution of brain swelling. To unravel this question, we monitored the evolution of ECM *in vivo* using high-field magnetic resonance imaging (MRI) with whole-brain coverage and have identified a distinct pattern of cerebral disease spread. Inflammatory disruption of the blood-brain-barrier and consecutive brain swelling initiates in the olfactory bulb and spreads from there along the rostral migratory stream—a neurogenic niche—deeper into the brain. When the brainstem is eventually reached, mice start to fall into a comatose state. Those findings correlate with previously published human MRI findings, which also show brain swelling of the brainstem in comatose children with cerebral malaria as well as early involvement of the striatum—recently recognized to serve neurogenesis in humans. Our study provides a novel link between neurogenic areas specifically permitting the spatiotemporal expansion of activated microglia, blood-brain-barrier disruption and consequent brain edema. Finally, the dominant role of the neurogenic axis in the transmission of inflammation may provide an explanation why children are more vulnerable to cerebral malaria.

## Introduction

Malaria remains among the most frequent and fatal infectious diseases worldwide. The WHO reported 207 million cases of malaria in 2013. Most of the 584,000 estimated deaths related to the disease occurred in children under 5 years of age [1] and were mainly a sequel of cerebral malaria (CM), the most severe complication of *Plasmodium falciparum* infection. CM patients show a rapid progression from headache and general malaise to hemiparesis, ataxia, unrousable coma and death occurring most frequently due to respiratory arrest [1, 2]. Children are significantly more vulnerable to CM compared to adults [3–5]. The reasons for this obvious age-dependent difference are unclear [6, 7]. There is consensus that severe brain swelling occurs at a late stage of the disease, and that it is a strong predictor of fatal outcome in both human and experimental CM [8, 9]. However, the exact pathological cellular and molecular mechanisms underlying CM are still unknown and it has remained poorly understood how exactly brain injury is confined in space and develops over time before fatal brain swelling ultimately evolves. Microvascular changes such as intravascular sequestration and endothelial adhesion of infected red blood cells to the endothelium of the cerebral microcirculation have been implicated in the development of severe brain swelling [2, 10, 11]. Microvascular plugging could result in impaired perfusion and consecutive ischemic cytotoxic brain edema [8, 12, 13]. An alternative concept has emerged positing that an exacerbated immune response is elicited within or against the host CNS and may be triggered by a “storm” of cytokines eventually leading to blood-brain barrier disruption (BBBD), vasogenic edema and brain swelling [14, 15]. Interestingly, human post mortem studies [11, 16–18] revealed the presence of host leukocytes at the site of parasite sequestration in the brain microvasculature after severe cerebral malaria. Also in malaria infection animal models, such as the *Plasmodium berghei* ANKA (PbA) model, a well established and widely adopted experimental system of CM (ECM) that reflects many features of human CM [19], leukocyte accumulation in the cerebral microvasculature is found consistently [20–23]. These observations suggest that apart from secreted cytokines, cellular inflammatory infiltration might also play an important role in CM by mediating an exacerbated immune host response targeted against the CNS [15]. Identifying trafficking pathways by which a potential inflammatory molecular and cellular response is mediated and progresses would lead to a better understanding of CM and would help to identify novel strategies of therapeutic intervention and diagnostic targets for early monitoring of beneficial effects of any such intervention.

In this study, we employed multimodal high-field MRI and contrast agents sensitive to early BBBD to identify specific routes of inflammatory brain infiltration in ECM. This enabled us to monitor *in vivo* with fine neuroanatomical detail the spatial occurrence and temporal progression of BBBD, brain edema and microvascular pathology. We identified the rostral migratory stream (RMS) as a specific route of microglial activation. These in vivo imaging findings allowed targeted histological sampling of the RMS and its connection to the olfactory bulb (OB) on sagittal brain sections. Quantitative and automated immunohistochemical analyses were then performed to analyze specific alterations of microglial and neuroblast cell populations along the RMS.

## Results

### Blood-brain-barrier disruption and edema initiate in the olfactory bulb and spread along the rostral migratory stream

To assess early blood-brain barrier disruption (BBBD) two different contrast agents were used: Gd-DTPA in the first group and Gadofluorine-M (Gf-M) in the second group. Gf-M is an experimental contrast agent binding to serum albumin that has been shown to detect subtle BBBD with higher sensitivity than the standard contrast agent Gd-DTPA in neuroinflammatory diseases [24]. The higher sensitivity of Gf-M despite its higher molecular weight is likely caused by its binding to extracellular matrix proteins after having crossed the disrupted BBB, in contrast to Gd-DTPA, which is not trapped locally in the extracellular space and therefore diffuses back more easily into the intravascular compartment after having crossed the impaired BBB [25]. Gf-M was therefore given early at day 7 post infection, when the RMCBS of all mice was still at 20, and also before the earliest Gd-DTPA changes had been detected in the previous experiments.

**Fig 1:**
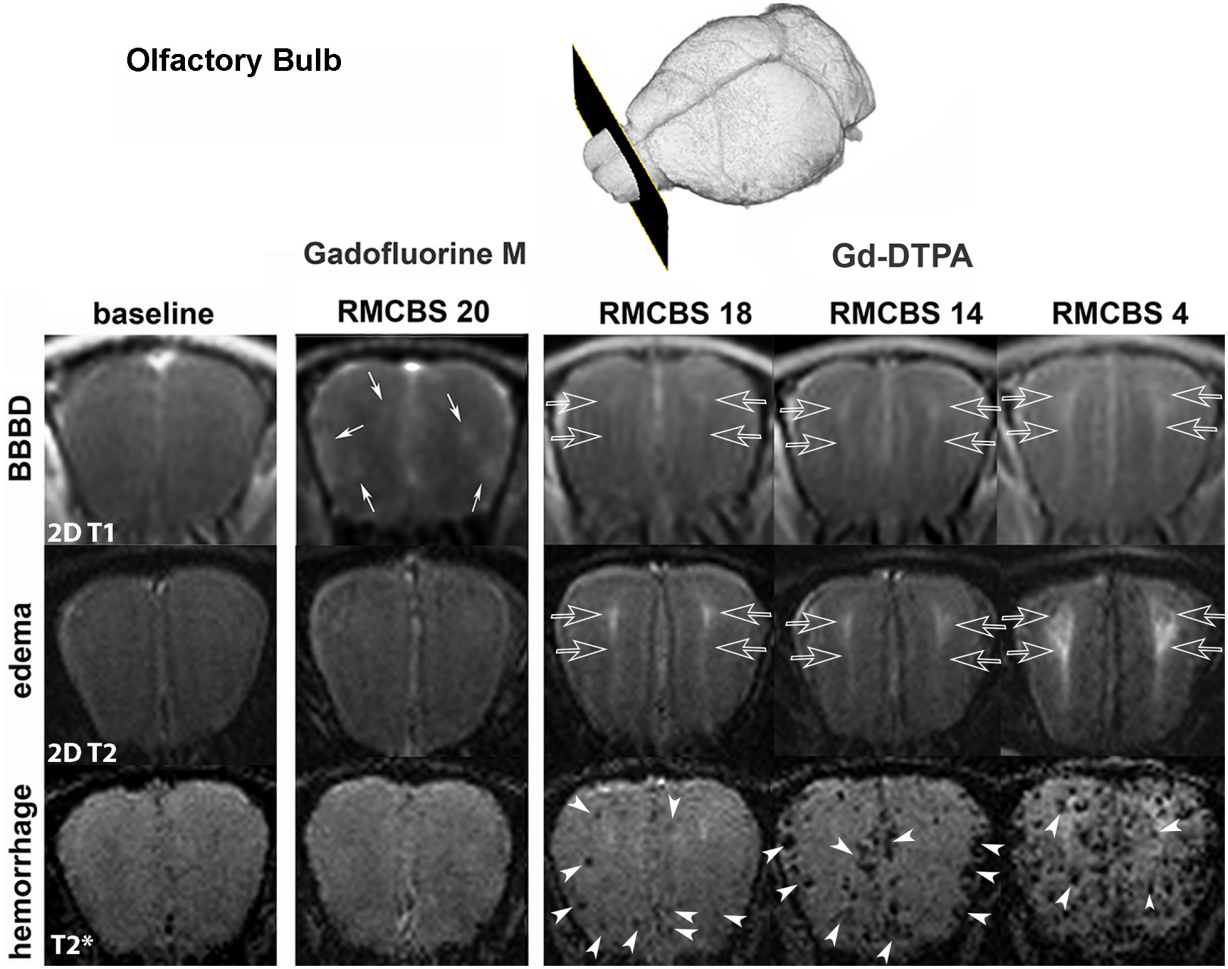
ECM initiates in the olfactory bulb. Upper row (blood-brain barrier disruption): Representative coronal 2D T1w- images at baseline (left column), after iv. Gadofluorine M (Gf-M, second column) and after Gadolinium-DTPA administration (third to fifth column); The high-molecular contrast agent Gf-M (bound to albumin) showed a multifocal extravasation pattern with predominance at the outer regions of the OB, when mice were clinically healthy = RMCBS 20 (small white arrows, second column). Before any moderate or severe clinical symptoms were evident (RMCBS 18) a diffuse extravasation pattern of the low-molecular contrast agent Gd-DTPA occurred and was mainly visible in the central region of the OB (parallel transparent arrows, third to fifth column). Middle row (edema): Representative 2D T2-weighted images (echo time = 22ms) showed normal signal at baseline (first column) and when multifocal Gf-M extravasation had already occurred in clinically healthy mice = RMCBS 20 (second column). When Gd-DTPA extravasation was evident in the central region of the OB, T2 signal also increased (parallel transparent arrows, third column) and continued to increase with progressive disease (parallel transparent arrows, forth and fifth column). Lower row (hemorrhage): Representative T2*-weighted images (echo time = 18ms) sensitively revealed microhemorrhages. At baseline (first column) and when multifocal Gf-M had already extravasated in clinically healthy mice = RMCBS 20 (second column) no microhemorrhages were detected. Before any moderate or severe clinical symptoms were evident (RMCBS 18) few microhemorrhages had occurred in the outer regions of the OB at similar locations of Gf-M extravasation (white arrowheads, third column). In moderate disease (RMCBS 14) microhemorrhages were still mainly located in the outer regions of the OB (white arrowheads, forth column). In severe disease (RMCBS 4) microhemorrhages had also occurred in the central regions of the OB (white arrowheads, fifth column). Mice of the two different groups are displayed. In the second column labelled "Gadofluorine M" one mouse of the second group is shown; in the columns denominated "Gd-DTPA" images of 2 mice of group 1 at different stages of severity are shown (baseline, RMCBS 14 and RMCBS 4 are from the same mouse, while RMCBS 18 is from another mouse).

After Gf-M injection the earliest sign of disease was a multifocal BBBD in outer regions of the OB, visible as multifocal T1-w bright, hyperintense lesions. This observation was made consistently in all mice receiving Gf-M, i.e. before any clinical signs became evident and before any signs of vasogenic edema or microhemorrhages could be detected (Fig 1). With further disease progression but still before any moderate/severe symptoms became evident (mean Rapid-Murine-Coma-and-Behavioral-Scale (RMCBS) = 18.5±1) microhemorrhages occurred in similar regions as prior multifocal Gf-M leakage, namely in the outer regions of the OB and were mostly located in the OB already during early disease. At the same time the earliest pathological changes with the low-molecular-weight contrast agent Gf-DTPA had occured. In contrast to early Gf-M leakage and microhemorrhages, Gd-DTPA extravasation (seen as bright image contrast) was first seen centrally. Also a T2 signal increase (seen as bright image contrast) could be detected in the central region of the OB on quantitative and on T2-weighted images, which is, together with BBBD, strongly indicative of vasogenic edema (Fig 1)

The evidence of early vasogenic edema beginning in the OB+RMS was further strengthened by quantitative imaging values: an increase in T2 relaxation time, derived from T2 relaxometry and increased apparent diffusion coefficient (ADC) values, derived from diffusion-weighted imaging. Both parameters indicate an increase in extracellular fluid, which accumulates during vasogenic edema formation.

**Fig 2:**
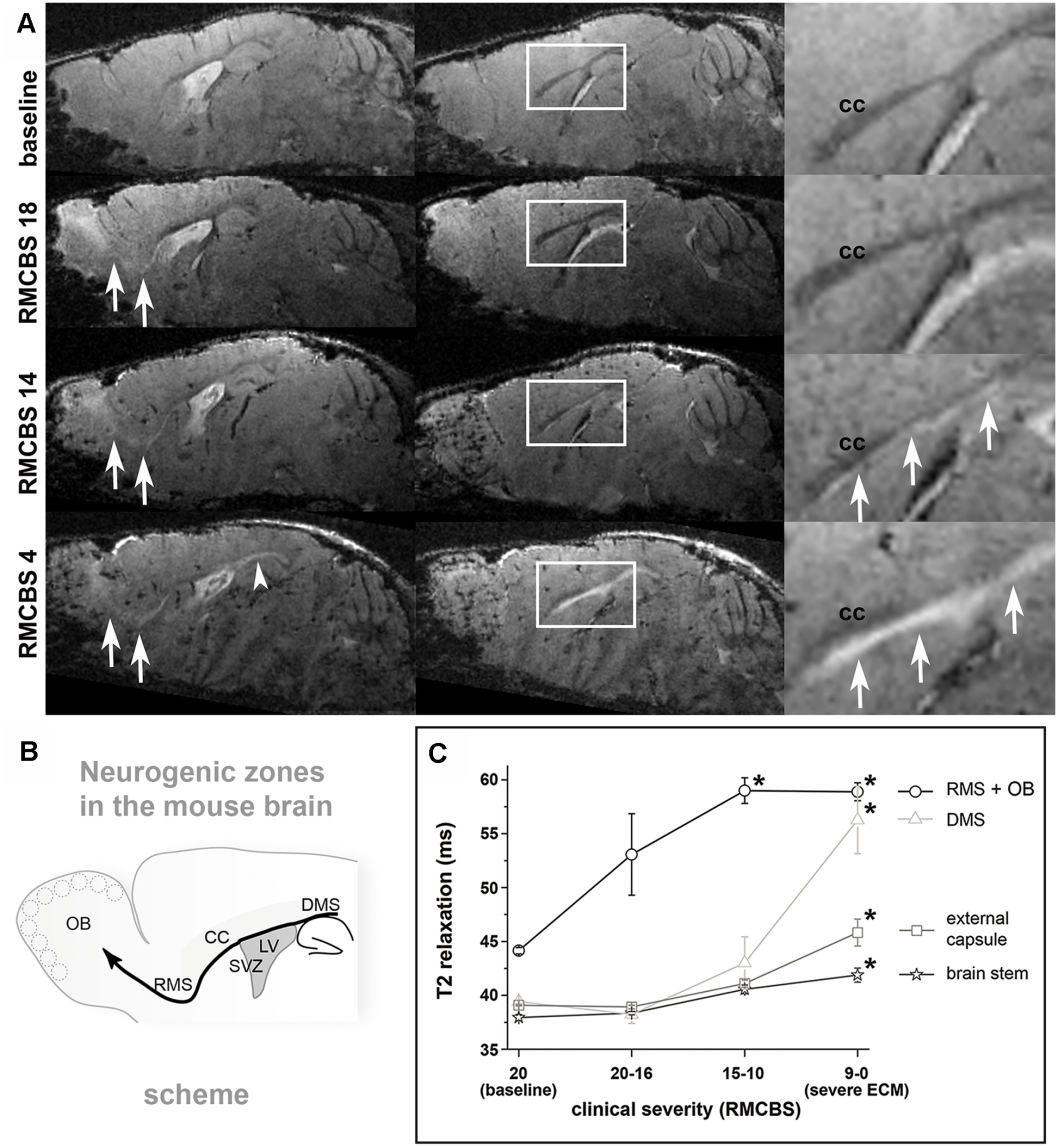
Edema spreads along the RMS in rostral-to-caudal direction. **A:** Edema, seen as hyperintense (bright on T2*-weighted images) signal, started in the OB (white arrows in left column of A) and with progressive disease (from top to bottom) continued to spread from there along the RMS to the subventricular zone of the lateral ventricles. At high magnification zooms (right column with zoom region indicated in middle column) the involvement of the subcallosal subventricular zone and the dorsal migratory stream is apparent, but does not include the corpus callosum (“cc”) per se (arrowhead in focused magnifications and solid white single arrow on lower image in left column). Rostral-to-caudal extension was associated very closely with disease manifestation and progression both defined by decreasing RMCBS (RMCBS values are denoted on left of A). **B:** Schematic drawing of neurogenic regions in the mouse brain is presented. Neuroblasts migrate in the direction of the thick solid black curved arrow from the subventricular zone (SVZ) along the rostral-migratory-stream (RMS) to the olfactory bulb (OB) or along the dorsal-migratory-stream (DMS) above the hippocampus towards the occipital cortex (short curved thin black arrow pointing to right margin of image). **C:** T2 relaxation time increased in the RMS+OB early in the disease, when mice did not show evident clinical symptoms and was significantly elevated in progressive disease (RMCBS 15–10 and 9–0 each with p<0.05 versus baseline as indicated by asterisk, solid black line and circles indicating data for RMS+OB). A neuroanatomical landmark indicating transition to severe ECM is involvement of the dorsal-migratory-stream (DMS) showing significantly elevated T2 times in RMCBS 9–0 (p<0.05, asterisk). At this stage, also further spread into the external capsule and eventually into deeper brain structures and the brainstem occured (all <0.05 for severe ECM with RMCBS 9–0).

In contrast to microhemorrhages, BBBD (evident as contrast extravasation of Gf-M and Gd-DTPA both assessed qualitatively) and vasogenic edema (evident as increase of quantitative T2 relaxation time and ADC) was not confined to the OB, but started to spread along the RMS, i.e. a migration route for immune cells and neuroblasts (Fig 2A and 2B). Immediately after clinical manifestation (mean RMCBS = 14.2±0.4), the increase of quantitative T2 and ADC (+33% / +29%) reached statistical significance in the OB+RMS compared to the baseline scan performed in infected mice before onset of blood stage infection (p = 0.049/p = 0.01). At the same time Gf-M and Gd-DTPA extravasation as well as a slight T2 and ADC increase were already visible in the dorsal migratory stream (DMS) and along the external capsule (EC), however, a statistical increase in these more posterior regions was not yet reached. When RMCBS scores dropped below 10 (severe ECM: mean RMCBS = 5±2.9) extravasation of Gd-DTPA had reached the brainstem and a significant increase of T2 in brainstem (+12%), DMS (+50%) and external capsule (+20%) was also evident (p = 0.034) (Fig 2C). ADC values of these regions showed a positive trend, but did not reach statistical significance.

### Olfactory bulb and cibriform plate

**Fig 3:**
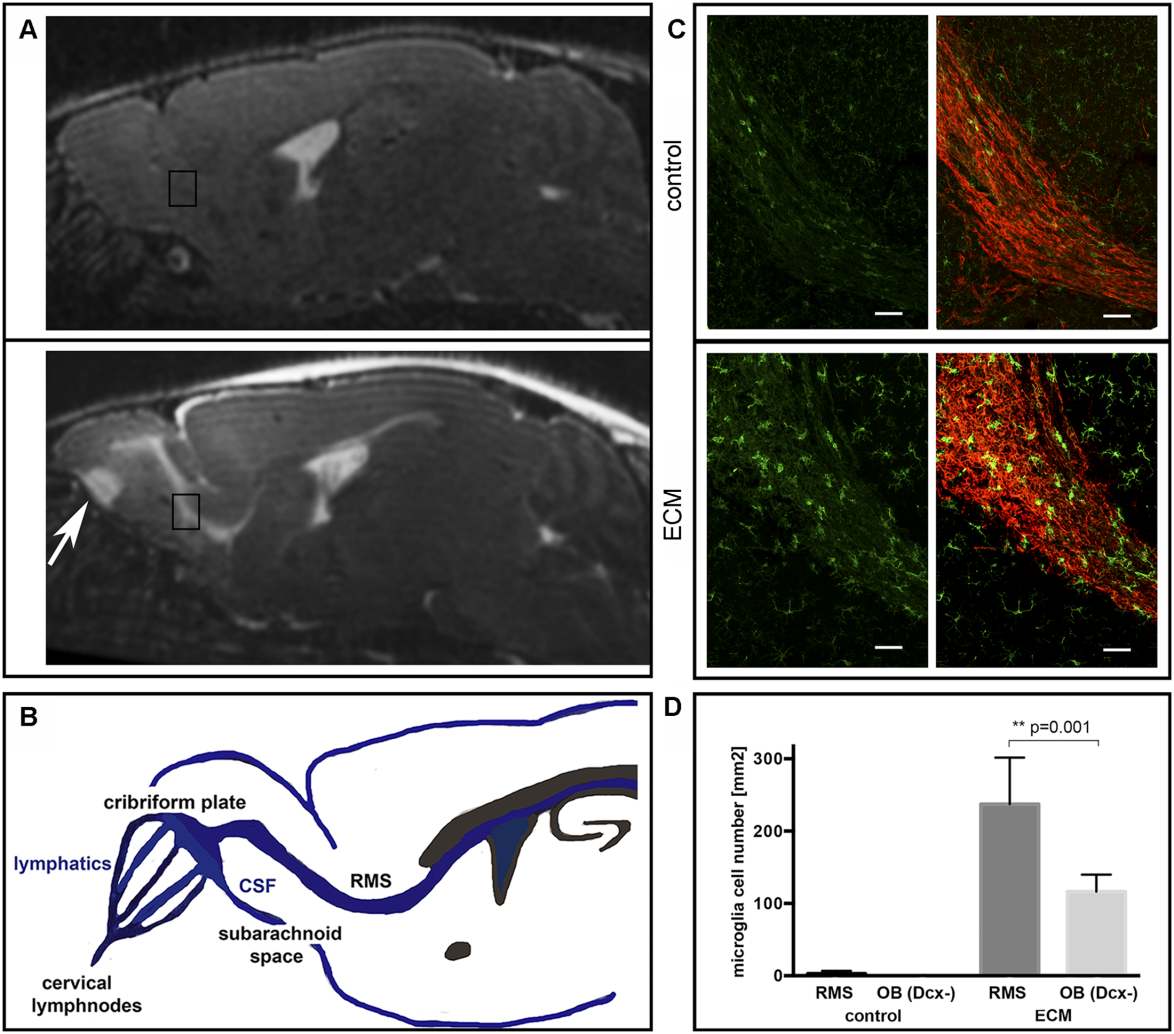
Microglial cells are closely associated with the rostral migratory stream. (A) On T2w images, the rostral migratory stream shows strongly hyperintense signal, indicative of vasogenic edema as a consequence of inflammatory BBBD. At the cribriform plate edema can also be appreciated (white arrow), emphasizing that the known anatomical connection of perivascular CSF spaces with the nasal lymphatics at the level of the cribriform plate is an important route of inflammatory infiltration in ECM (B). Inserted squares in panel (A) refer to the anatomical location of the co-immuofluorescent images in panel (C). Co-immunofluorescent staining of Iba-1 (green) and doublecortin (red) on sagittal brain sections through the RMS elbow in a representative control and an ECM mouse. In the ECM mouse, a clustering of activated microglial cells is visible along the RMS with a significantly increased number of activated microglial cells along the RMS (doublecortin positive regions) compared to a lower microglial cell number in the vicinity around the RMS in doublecortin negative regions. (D). Bars indicate 50μm.

Interestingly, in the OB, BBBD (extravasation of Gf-M and Gd-DTPA) and edema (increase of T2 and ADC) occurred in direct vicinity to the cribriform plate in all mice (Fig 3A). This finding represents very early visualization of ECM-triggered BBBD and vasogenic edema in a neuroanatomical region where the perivascular space of the RMS is directly connected to the nasal lymphatics at the level of the cribriform plate and where the blood-brain-barrier is leakier than in other brain regions e.g in the pons or the cortex [26] (Fig 3A and 3B).

### Activated microglia are closely associated with the rostral migratory stream–an immune cell and neuroblast trafficking route with access to the nasal lymphatics

**Fig 4:**
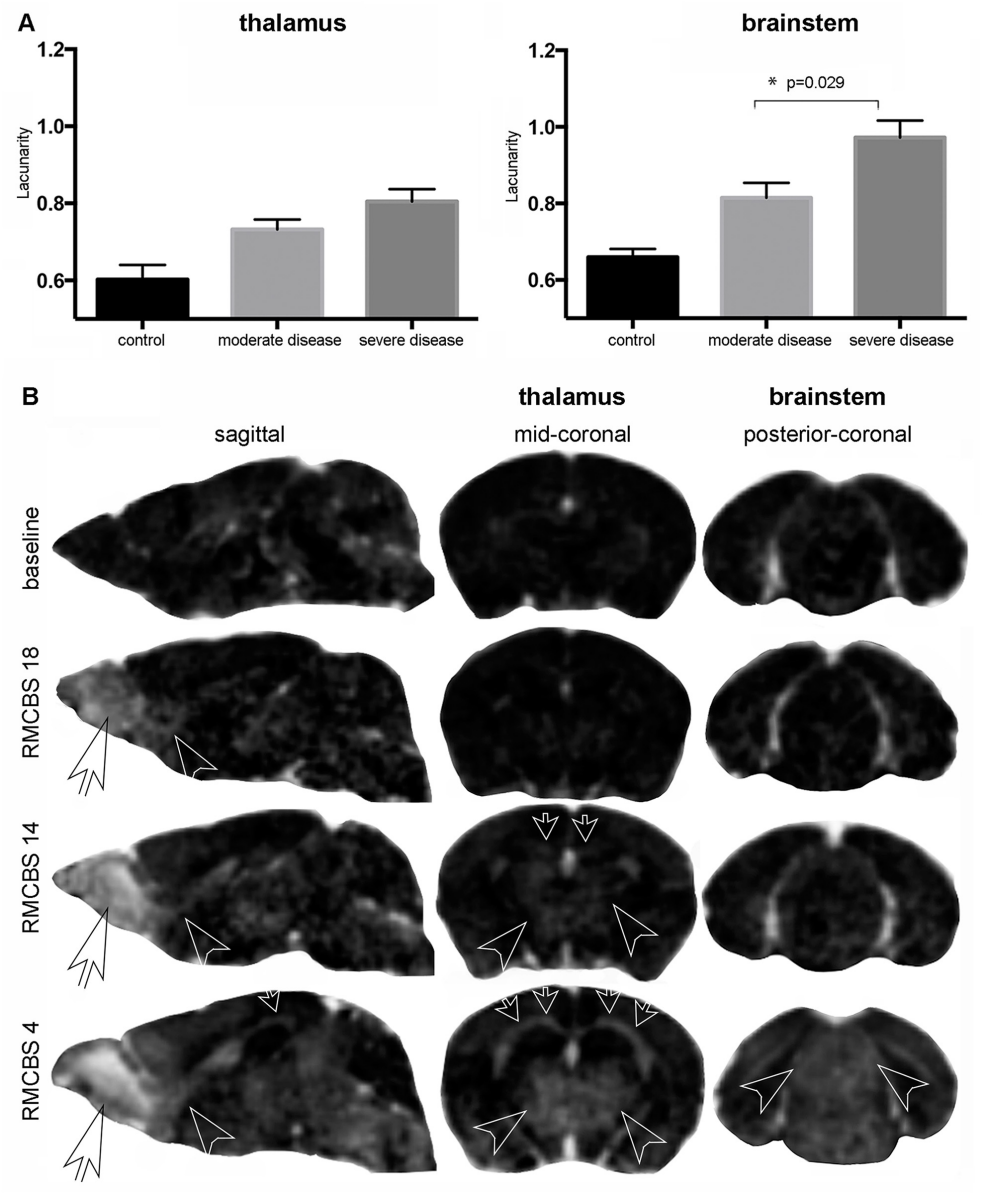
In severe disease edema extends to the brainstem. A: T1 image relative variation of contrast by subtracting pre- from post -contrast images (after i.v. Gd-DTPA). Contrast enhancement indicative of BBBD is seen early on in the OB and anterior aspect of RMS (white transparent arrowheads in left column and second row: RMCBS 18, sagittal orientation). With increasing disease severity (third and fourth row: RMCBS 14 and RMCBS 4) contrast enhancement increases and includes the RMS completely (white transparent arrowheads left column, third and fourth row), extending towards the DMS (small white transparent arrows left column and middle column), deep grey matter and brain stem. Involvement of deep grey matter and brain stem can be better appreciated on coronal image sections (middle and right column, coronal orientation). B: Lacunarity values at the level of the thalamus and the brainstem are presented. During moderate (n = 5) and severe disease (n = 6) lacunarity values in the thalamus were not significantly different. In the brainstem, however, lacunarity increased significantly in severe disease compared to moderate disease.

On histological sagittal sections through the RMS in control mice, no activated microglial cells (visualized by the microglial cell marker Iba-1) were present along the RMS (visualized by the neuroblast marker doublecortin). In contrast, activated microglial cells were consistently evident in ECM mice and they were in very close proximity to the RMS (Fig 3C). The microglial cell number per mm^2^ associated with the RMS (237.4 ± 22.7 mm^2^) was significantly higher than in doublecortin negative regions adjacent to the RMS (116.4 ± 8.3 mm^2^; p = 0.013; Fig 3D). Microglial cells adjacent to the RMS were found in a less activated state, than microglial cells directly associated with the RMS emphasizing that the inflammatory spread occurs along the RMS. Along the RMS microglial cells mainly showed an active or transitional activation state, while in the brain parenchyma the withdrawal stage dominated, further indicating an earlier activation of microglial cells along the RMS. To further analyze parenchymal microglial activation the quantitative fractal analysis measure ‘graphical lacunarity’ was used [27]. Graphical lacunarity (Λ) captures the different morphological states of microglial cells from a resting to an activated state by analyzing the gappiness of an image and the degree of inhomogeneity in the object (S1 Fig). In the brain parenchyma graphical lacunarity increased with increasing activation of microglial cells. Significantly higher microglial activation in the brainstem in severe disease was revealed compared to moderate ECM (Λ = 0.97±0.05 vs. 0.81±0.04 p = 0.029), but not in the thalamus (Λ = 0.80±0.03 vs. 0.73±0.02; p = 0.12) (Fig 4A). This quantitative morphological measure of microglial activation correlated with imaging and clinical findings since only in severe disease BBBD (Fig 4B) and a significant increase of quantitative T2 were evident in the brainstem, when mice reached a comatose state.

### Neuroblasts in the RMS exhibit altered morphology and migratory pattern

**Fig 5:**
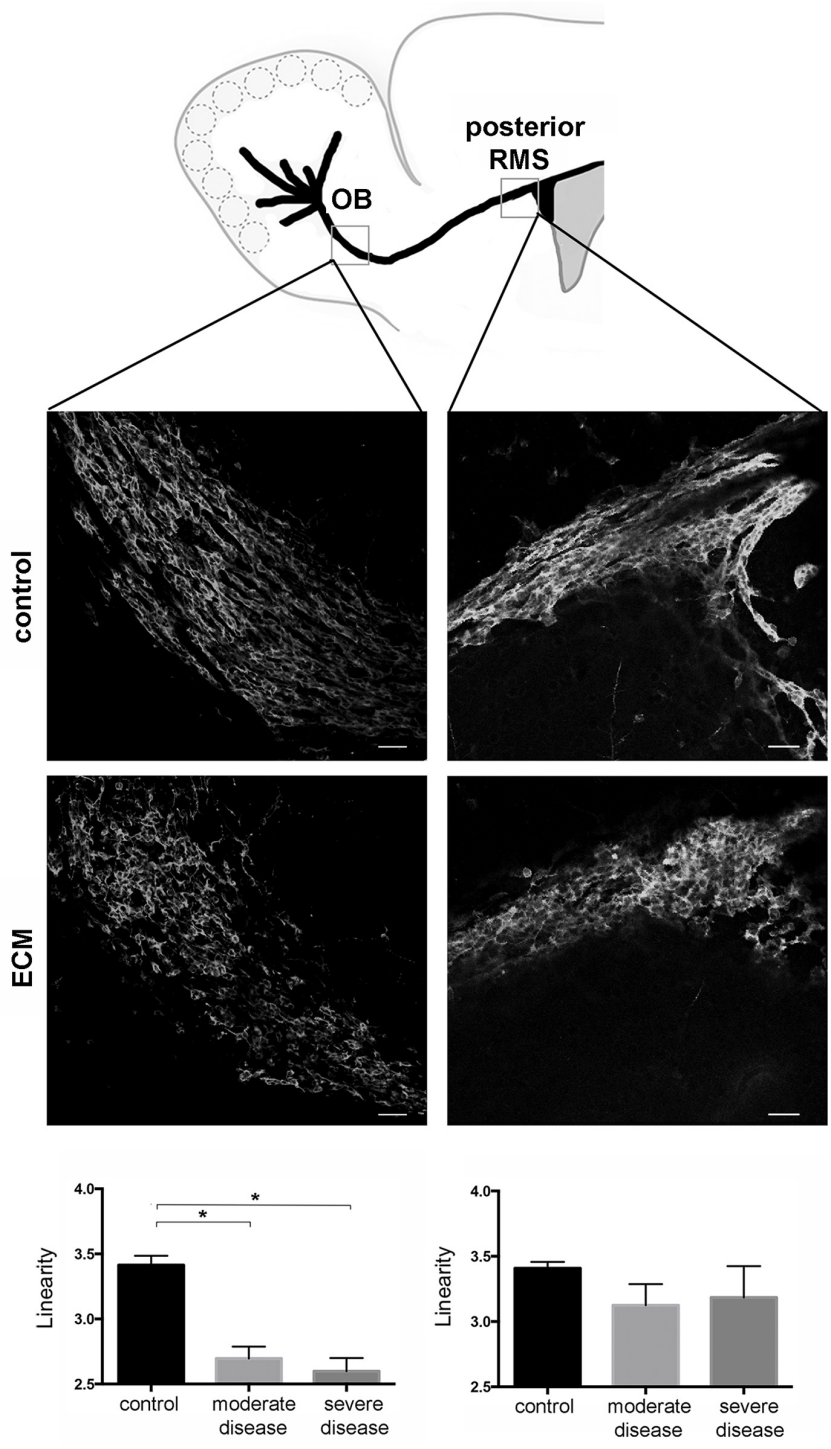
Severe disturbance of neuroblast chain migration along the RMS. Immunofluorescent staining against doublecortin on sagittal brain sections of a representative control and an ECM mouse at the level of the RMS elbow and posterior RMS (illustrated in schematic drawing) as well as bar charts quantitatively assessing the linear migration pattern of neuroblasts (controls n = 3, moderate disease n = 5, severe disease n = 6). A linearity index was calculated to analyze morphological changes of neuroblast alignment (quotient of major axis length and minor axis length). Compared to controls, neuroblasts in ECM mice show severely altered morphology. The typical chain migration pattern and the linear appearance deviate when compared to controls and is more pronounced in the OB, but can also be detected in the posterior RMS (Bars = 50μm).

As activated microglial cells were primarily detected within the RMS, morphology of neuroblasts themselves was assessed. Immunohistochemical staining for doublecortin, a marker for neuronal precursor cells, revealed altered neuroblast morphology along the RMS in all ECM mice. In moderately and severely sick ECM mice neuroblasts in the OB showed a severely abnormal pattern deviating clearly from their usual spatial organization: they displayed curved processes with random outward orientation instead of straight leading processes, which is consistent with severe misalignment, i.e. diverging from their typical linear arrangement (Fig 5).

Morphological changes of neuroblast shape and geometry were thereby quantified using an index of linearity, which was calculated as the quotient of major axis length and minor axis length of migrating neuroblasts (S2 Fig). Under physiological conditions neuroblasts migrate in chains displaying a linear alignment approaching values of four, while under pathological conditions values steadily decrease.

The linearity index of analyzed neuroblasts in the OB compared to uninfected control mice (3.4 ± 0.1) was significantly decreased in moderately (2.7 ± 0.1; p = 0.001) and severely sick mice (2.6 ± 0.1; p = 0.001). In the posterior RMS morphology of neuroblasts in ECM was also altered, even though the difference in linear alignment in moderately (3.1 ± 0.2) and severely sick (3.2 ± 0.3) ECM mice compared to controls (3.4 ± 0.1) did not reach significance (Fig 5).

Altogether, these observations indicate that the inflammatory cellular response is associated with altered neuroblast chain migration in the RMS and initiates in the OB.

### Temporal and spatial evolution of microvascular pathology recapitulates that of BBBD and edema

**Fig 6:**
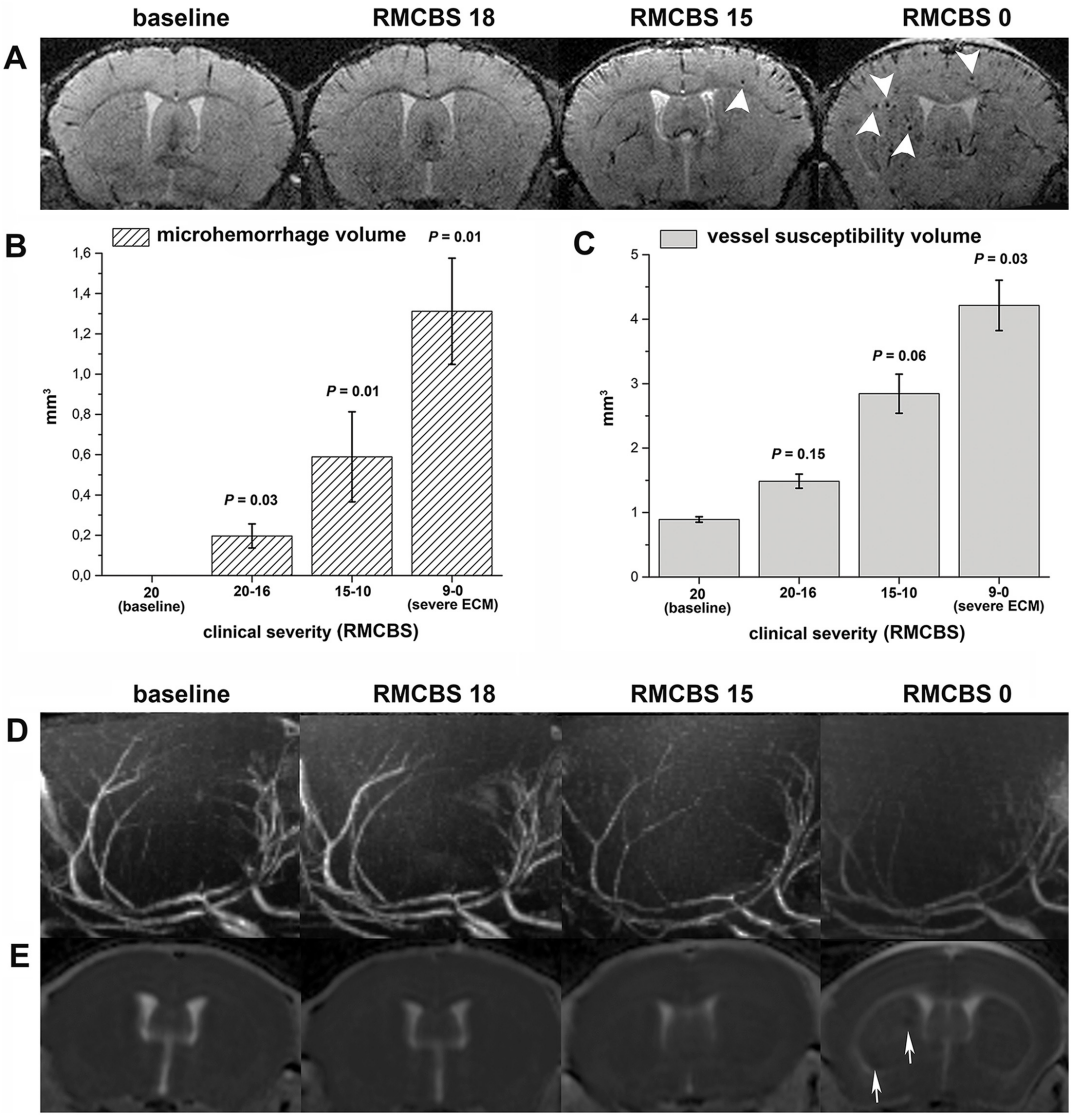
Microvascular pathology. T2* (A, coronal orientation), maximum-intensity-projection reconstructions of time-of-flight angiographic images (B, coronal orientation) and ADC maps (C, coronal orientation) at different RMCBS grades are presented (denoted on top). Microvascular pathology in ECM increases with disease severity and is paralleled by the occurrence of microbleeds (arrowheads, row A). It is also paralleled by increasing vessel density on T2* images consistent and by vessel rarification on TOF angiographic images. Both these latter findings are indicative of slow flow or microvascular sequestration in severe disease (row B). Ischemic infarctions, if present, were seen in very severe disease only and were confined to restricted regions of watershed/borderzone regions, which suffer first from low cerebral perfusion (arrows, row C). On ADC images edema was also seen underneath the corpus callosum and later along the external capsule as hyperintense signal alteration. Volumetric quantification of susceptibility vessel volumes (D) and microhemorrhage volumes (E) are displayed in the bar charts both plotted as a function of disease severity (RMCBS on x-axis).

On baseline scans no microhemorrhages were evident. Before clinical manifestation, but after BBBD (mean RMCBS = 18.5±1) microhemorrhages occurred in the peripheral regions of the OB, mainly in the periglomerular and mitral cell layer (Fig 1), and very few microhemorrhages were present also along cortical vessels with a total microhemorrhage lesion volume of 0.20±0.13 mm^3^ (significantly elevated compared to baseline; p = 0.03). At moderate disease severity (RMCBS = 14.2±0.4) the total microhemorrhage volume increased further to 0.59±0.5 mm^3^ (p = 0.01) and microhemorrhages were present also in the basal ganglia (Fig 6A). Finally, when ECM reached a severe stage (mean RMCBS = 5±2.9), microhemorrhage volume further increased to 1.3±0.74 mm^3^ (p = 0.01) (Fig 6B). Microhemorrhages were confirmed on HE sections, showing the highest load of microhemorrhage volume in the OB of ECM mice with occurrence of microhemorrhages also in the cortex, basal ganglia, cerebellum, white matter and brainstem (S3 Fig).

With increasing disease severity also an increasing volume of pathological vessel susceptibility became obvious. Under physiological conditions T2* images display venous vessels. Under pathological conditions an increase in susceptibility volume can result from slow flow, decreased oxygenation or vessel obstruction/thrombosis. Increasing vessel susceptibility contrast was evident primarily in penetrating cortical and deep vessels, and a gradient that increased from the brain surface along the cortical perforators towards the white matter was apparent. The total volume of pathological vessel susceptibility contrast was highest in severe disease (4.21±1.10mm^3^) compared to baseline (0.89±0.06mm^3^, p = 0.03). The total pathological susceptibility volume of mice before any moderate/severe symptoms became evident (1.49±0.21mm^3^; p = 0.15) and of mice with moderate symptoms (2.84±0.68mm^3^; p = 0.06) was also increased, but did not reach statistical significance (Fig 6C).

In close correspondence to pathological vessel susceptibility contrast with progressive ECM, progressive vessel rarification and reduction of vascular lumen was detected by means of high-resolution MR-ToF-angiography (Fig 6D). Vessel rarification became obvious with disease manifestation (Fig 6D, mean RMCBS = 14.2±0.4). In severe disease (mean RMCBS = 5±2.9), there was a strong decrease of vascular lumen in all mice (Fig 6D).

Together these findings are highly indicative of low blood flow and microvascular sequestration in severe disease.

Ischemic lesions, evident as a decrease in ADC, were never seen before day 9 after infection and were never present during early or moderate ECM. Only in 22% of mice with severe ECM few cerebral ischemic lesions were detected by diffusion-weighted imaging. These few lesions occured in watershed areas known to be supplied by distal penetrating branches from two opposite directions: cortical and basal lenticulostriate perforators and suffer first from low cerebral perfusion [28].

## Discussion

In this study we monitored *in vivo* and with unprecedented neuroanatomical detail where edema in cerebral malaria initiates and how it spreads deeper into the brain. The strikingly close association of inflammatory BBBD with the OB+RMS axis and its rostral-to-caudal ascent within these distinct regions strongly suggest that eventual structural brain injury in ECM results from an exacerbated immune host response arising from the OB+RMS axis, which represents a key route for the CNS immune response and CNS neurogenesis. This specific immune cell and neuroblast pathway has also been implied in other, non-infectious triggers of neuroinflammation [29].

Our observations from MRI at high magnetic field strength, enabling a structural resolution of up to 80 x 80 x 80 μm and whole-brain coverage are in good agreement with results obtained by another study using intravital microscopy to observe BBBD in focal regions of the cortex of ECM mice (field-of-view ~150x150μm^2^, depth of penetration ~50μm) [30]. These authors similarly reported that the degree of BBBD was closely associated with neurological signs suggesting that opening of the BBB is related to clinical disease progression [30]. In addition, they observed that ECM mice exhibited platelet marginalization followed by extravascular fibrin deposition, and extensive vascular leakage at the level of the postcapillary venules, which represent a key element of the so-called neuroimmunological BBB. Previously the importance of the neuroimmunological BBB has been described in autoimmune diseases such as multiple sclerosis and its experimental model autoimmune encephalitis (EAE), where immune cells enter into the CNS via the postcapillary venules: the first step involves cellular inflammatory infiltration into the perivascular space surrounding the postcapillary venules and the second step entails infiltration across the glia limitans into the brain parenchyma [31]. Yet other intravital microscopy studies, analyzing leukocyte trafficking in the cortical microvasculature (focal, cortical sampling volume ~150x150μm^2^/300x300μm^2^), revealed that CD11b+ macrophages and CD8+ T cells are present in the perivascular space [32–34]. Also perivascular CD8+ cells showed a more arrested phenotype in mice developing EMC, emphasizing the importance of the perivascular space as immunological compartment in the development of ECM [34]. These important previous observations were made on the microscopic cellular level, with focal cortical field-of-views reaching a depth of penetration of about 50μm into the cortex *[30, 32, 33]*. We now add information based on *in vivo* studies supporting the notion that very early inflammatory BBBD and consequent perivascular spread of vasogenic edema arise at the level of the OB+RMS. Only with progressive clinical disease, a further rostral-to-caudal extension of inflammatory BBBD and edema occurs ultimately reaching the brainstem in severe disease.

Our findings provide strong evidence that the OB+RMS, which represents a structurally and functionally unique axis of immune and neuroblast trafficking, is the structure permitting and transmitting the inflammatory response of ECM deeper into the brain.

We further corroborated this hypothesis by showing histologically that microglial cells specifically appeared in a more activated state when they were localized inside or in the immediate vicinity to the OB+RMS (= doublecortin positive regions), and in a less activated state or without signs of activation when they were localized only in adjacent brain areas along and with greater distance to the RMS (= doublecortin negative areas). This observation is consistent with an early specific inflammatory activation of microglial cells strictly associated with the OB+RMS axis. In fact, microglial cells are considered to be an integral part of the neurogenic niche [35] and have been reported to cluster in the perivascular space if exposed to triggers such as e.g. fibrinogen [36] that also extravasates in ECM. The exact anatomical association of BBBD and edema with the OB+RMS neurogenic axis suggests that inflammation in ECM uses this perivascular neurogenic niche to infiltrate and extend deeper into the brain. Before clinical signs were evident, BBBD and edema were present along the OB+RMS. Notably, in sagittal high-resolution sections of the OB (Fig 3c, white solid arrow), it was apparent that edema initiated and predominated at the base of the OB visualizing the exact location of its connection to the cribriform plate. Exactly at the cribriform plate, cerebro-spinal-fluid (CSF) and perivascular fluid connect and exchange with the nasal lymphatic system, which is interwoven with the cribriform plate [37, 38]. The perivascular space of the OB+RMS represents the bridge between CSF of the lateral ventricles and the cribriform plate as gate to the nasal lymphatic system. We therefore speculate that inflammatory BBBD, which is triggered first in the OB, hinders the drainage of perivascular CSF towards the nasal lymphatics promoting the retrograde expansion of inflammation and vasogenic edema along the RMS.

This novel finding suggests that the dynamics of perivascular bulk flow, which has been denominated by Iliff et al. the *“glymphatic” system* of the brain [39, 40], may play a key role in the rapid inflammatory spread of the disease along the OB+RMS axis, and from there into deeper brain areas. CSF represents a major reservoir for complement factors and immune cells in the CNS that accumulate in disease [41, 42]. The perivascular space can consequently act as an important immune compartment during vasogenic edema formation enabling a direct interaction of leukocytes and endothelial cells with pericytes, astrocytes and microglia [43, 44]. In addition cytokines can be released into the perivascular CSF and spread via the perivascular space through the brain, potentially explaining the rapid spread of disease in ECM [45]. We therefore hypothesize that antigen presentation to perivascular immune cells through e.g. antigen-presenting endothelial cells or infected red blood cells present in the perivascular space triggers an antigen-mediated immune response [34, 46].

We detected the earliest sign of ECM in the OB and not in other anatomical regions by multifocal leakage of Gadofluorine-M, pointing to an initiation of the immune response in the OB. Already in the healthy brain, the olfactory bulb and the rostral migratory stream show an increased BBB leakiness provided by a specialized vasculature with thinner BBB than in other regions of the brain [26, 47]. Firstly, a dense vascular network is present along the RMS, which is surrounded by a similarly dense network of perivascular spaces. This particular structural feature forms a scaffold for neuroblasts, which migrate in the perivascular space of this vascular network [48–50]. Secondly, VEGF is constantly expressed, regulating not only the growth of neuroblasts, but also of blood vessels and renders newly formed vessels more permeable [47, 49, 51]. These distinctive structural features of the microvasculature of the OB+RMS might explain why specifically the OB+RMS axis permits and transmits the inflammatory BBBD of ECM. However, we cannot exclude completely that a less pronounced inflammatory reaction possibly occuring in other regions may have evaded imaging detection by the MR methods used here.

Recently, others have also identified the OB as an important structure in the spatiotemporal progression of ECM [52]. Two-photon microscopy showed parasite accumulation and occlusion of trabecular small capillaries in the OB followed by microhemorrhages. In our study we substantially extend these findings by identifying the RMS+DMS as a structurally and functionally specific axis of ECM spread connecting the perivascular space of the OB with more caudal brain areas and particularly the subventricular and subcallosal zones of the ventricular organ.

Interestingly, the RMS has recently been identified also in experimental autoimmune encephalitis (EAE) as a central immune cell trafficking pathway [29]. In fact, in EAE it was shown, that blocking immune cell trafficking by a spingosine-1-phosphate receptor (S1PR) inhibitor (fingolimod) effectively protected the brain against EAE [29]. A similar scenario can be envisaged also for ECM. Interestingly, in ECM the same S1PR inhibitor was shown to prevent BBBD and the occurrence of consecutive clinical symptoms, even though the immune responses in EAE and ECM differ [30, 32]. These findings, taken together with our study in which we directly observed microglial activation and BBB disruption along the OB+RMS axis *in vivo*, strongly indicate that also in ECM the host immune response of the CNS is specifically mediated by antigen presenting cells of the RMS.

In contrast to BBBD and vasogenic edema, imaging signs of microvascular pathology did not precede but follow clinical symptoms. Ischemic lesions for example occurred only in very severe disease and in watershed areas of the brain. Microhemorrhages were predominately seen in the OB and may have been caused by venous outflow obstruction at the level of the postcapillary venules [32, 33]. In accordance with signs of venous outflow obstruction or microvascular plugging, pathological vessel susceptibility volume also increased with progressive disease. This increase in pathological vessel volume can be partly caused by higher parasite loads, because hemozoin, which is produced by the parasite also shows paramagnetic properties [53] or by slow or no flow, which causes susceptibility effects in vessels. Altogether, microvascular pathology on MR imaging was less associated with clinical symptom initiation and progression of vasogenic edema on MR imaging. This observation indicates that microvascular sequestration or slow flow in cortical microvessels, microhemorrhages and watershed infarcts occur secondarily after BBBD and vasogenic edema.

The main findings of our study, identifying the OB+RMS, a neurogenic niche and immune cell trafficking pathway, as axis permitting and transmitting inflammatory BBBD and vasogenic edema into the brain in ECM may also provide a plausible explanation for the preferential susceptibility of children to CM: The subventricular region extending to the rostral- and dorsal-migratory-streams is a neurogenic niche containing corridors for migrating neuronal precursor cells whose neurogenic capacity and migratory routes decline during infancy [54]. Also the local microvasculature and astrocyte organization of neurogenic niches changes from birth to adulthood. Blood vessels and astrocytes are aligned with the RMS in the adult [55], while in the neonate brain they are also located radially outside the RMS and cover a greater surface of the corpus callosum [50]. Additionally a glial sheath around the RMS is not yet found in neonates [56]. Since vessels and astrocytic processes serve as a scaffold for neuronal migration, this organization of the neurogenic niche provides a more permissive environment for parenchymal migration of neuroblasts in neonates compared to adults [50, 57]. The spread of inflammation in cerebral malaria may therefore be facilitated by a distinct morphological microvascular and astrocytic organization of the perivascular neurogenic niche in the young providing a permissive environment not only for neuronal migration, but also for inflammation.

We provide first evidence that neuroblasts are pathologically altered in ECM along the rostral migratory stream and with predominance in the OB. This observation could be made already in moderate disease indicating that neurogenesis itself is altered early during ECM. An increasing body of evidence suggests that significant crosstalk between neuroblasts and leukocytes may foster inflammation probably via specific yet unidentified cytokines having a regulative role on the proliferation of neuroblasts and on leukocytes [58]. The active role of neuroblasts is further emphasized by the failure of immune cell recruitment in case of RMS ablation [29].

Interestingly, an MRI study in children suffering from CM showed edema in the striatum in 84.2% of cases. The striatum was recently identified to serve neurogenesis in humans and is easily recognizable by MRI [59, 60]. MRI findings in adults with CM were clearly different showing striatal involvement only in 21% [59, 61]. Altogether our results suggest that ECM is pathoanatomically distinct from human CM in that the OB+RMS axis is central in the manifestation and progression of ECM, while the striatum may be a counterpart structure in humans involved early in pediatric CM. This anatomical difference, however, does not exclude a close functional similarity between ECM and human CM, since both anatomical areas, the OB+RMS and the striatum respectively, represent regions serving neurogenesis. Therefore, we speculate that areas of neurogenesis may also be preferentially involved in human CM, especially in children, and that they may also be important in regulating the inflammatory CNS response in human CM. Furthermore, neuroblasts in human CM may become similarly altered through the cerebral inflammatory response as observed here in ECM. This could add to a better understanding of the neurological sequelae from which CM survivors commonly suffer. These could be a result of or aggravated by a disturbance of the capacity of the brain for neurogenesis [62].

Beyond the involvement of neurogenic areas in both human and experimental CM further similar imaging features of CM and ECM are evident. In both species, secondary cerebral infarcts occur in watershed areas, white and grey matter are affected in a similar fashion and brain swelling involves the brainstem in severe disease, explaining the comatose state.

We therefore consider the ECM model a valid model for cerebral malaria as long as the specific biology and pathogenesis of the parasite species as well as anatomical and immunological differences of humans and rodents are taken into consideration.

In conjunction with previous evidence on the cellular microscopic level identifying the neuroimmunological postcapillary blood-brain-barrier as a target of an inflammatory immune host response in ECM [30, 32, 33], and with MRI findings in human adult and pediatric patients indicating an age-dependent effect of striatal involvement [59, 61], we now may provide the unifying hypothesis that cerebral malaria depends on a permissive environment generated by the perivascular neurogenic niche, which carries an exacerbated immune host response into the CNS namely along the OB+RMS axis in rodents and possibly along the striatum in humans.

## Materials and Methods

### Ethics statement

All animal experiments were performed according to FELASA category B and GV-SOLAS standard guidelines and approved by the local German authorities in Karlsruhe (Regierungspräsidium Karlsruhe, Germany, Approval number 35–9185.81 G-258/12).

### Murine malaria model

ECM was induced with the *Plasmodium berghei* ANKA (*Pb* ANKA) parasite in inbred 6–8 weeks old female C57BL/6J mice (Janvier Labs, France). *Pb* ANKA sporozoites (SPZ) were isolated by dissection of salivary glands from female *Anopheles stephensi* mosquitoes at day 18–21 post infection. In a first group (n = 16) infections were performed either by intravenous (i.v.) injections of 3x10^4^ SPZ in a total volume of 100μl sterile PBS (n = 8), or by subcutaneous (s.c.) injection (n = 8) [63]. This group of 16 mice was intended for longitudinal follow-up by clinical evaluation of symptoms and imaging. 10 out of these 16 mice could be included into the final data analysis (3 mice had to be excluded because they died of fulminant ECM with very rapid onset before any pathological MRI could be acquired; 3 mice did not develop symptoms of progressive ECM). In a second group, 5 additional mice were infected i.v. to evaluate earliest imaging signs of inflammatory brain infiltration with a specific contrast agent known to be more sensitive to BBBD (Gf-M) than the conventional contrast Gf-DTPA, which was employed in the first group. Mice of the second group were injected with Gadofluorine-M were sacrificed at the intermediate stage of disease (RMCBS 10–15) and used for histological analysis. Brains of three healthy female C57BL/6J mice were used as controls for histological analysis.

### Behavioral testing by rapid-murine-coma-and-behavioral-scale

For clinical evaluation, malaria-infected mice were assessed for ten parameters of cerebral symptoms according to the Rapid-Murine-Coma-and-Behavioral-Scale (RMCBS) [64]. RMCBS testing was performed immediately before MRI imaging.

The time of ECM onset relative to the time point of infection shows natural variation. For this reason, infected mice were grouped according to their RMCBS scores: 1) RMCBS 16–20 (before clinical manifestation; day 7.75±1.0 after infection), 2) RMCBS 10–15 (moderate ECM; day 7.8±1.1), 3) RMCBS 0–9 (severe ECM; day 8.3±0.8). Measures of seizure activity, which is also described in ECM [65], such as EEG recordings were not obtained.

### Experimental MRI protocol

MRI was performed on a 9.4 T small animal scanner (BioSpec 94/20 USR, Bruker Biospin GbmH, Ettlingen, Germany) using a volume resonator for transmission and a 4-channel-phased-array surface receiver coil. Anesthesia was induced per inhalation using 2% and maintained with 1–1.5% isoflurane. Animals were placed prone in fixed position monitoring body temperature and respiration.

In the first group the spatiotemporal progression of ECM was followed *in vivo*. MRI baseline scans were performed of infected mice before the onset of blood stage infection at day 1 or 2 after injection of infectious SPZ. These very early time points were always before the occurence of any clinical or pathological changes of ECM. The baseline scan served as intraindividual control as quantitative measurements of intraindividual measurements are more precise than interindividual measurements.

A second scan was performed at day 6 or 7 post infection before neurological symptoms occurred with the intention to detect subclinical early changes before ECM manifestation. Thereafter, in ECM mice the timing of further MRI scans depended on the intensity of ECM progression: subsequent scans were performed when mice developed clinical deterioration, i.e. when RMCBS fell below 16 (moderate ECM) or 10 (severe ECM). The MR sequence protocol is specified in detail in table 1. It included T1-, T2-, diffusion- and high-resolution T2*-weighted imaging (80μm isotropic resolution), T2 relaxometry, time-of-flight angiography (ToF) and T1- weighted imaging after contrast agent injection (0.3mmol/kg Gadolinium-DTPA (Gd-DTPA)). Gadofluorine-M (Gf-M) (0.1mmol/kg) as another Gadolinium compound used exclusively for experimental purposes was employed in the second group to increase sensitivity for early inflammatory BBBD compared to Gd-DTPA [24]. Gf-M binds to serum albumin and has a plasma half-life of 24h. In order to detect very early changes we injected the more sensitive contrast agent Gf-M at day 7 post infection as in the first group the earliest Gd-DTPA extravasation had occurred at day 7 and approximately 4 hours after injection. All mice of the second group (n = 5) were clinically healthy with an RMCBS = 20. Imaging was performed 4h after Gf-M injection, when mice were still scored with an RMCBS = 20. The imaging protocol carried out in the group receiving Gf-M included 3D T1-, T2 and T2*-weighted sequences. A second scan with the same protocol was performed when moderate disease had developed (10.8 ± 1 hours after injection of Gf-M) to confirm imaging changes of intermediate disease as brains were consequently used for histological analysis of intermediate disease (n = 5).

**Table 1:** Imaging protocol.

MRI protocol	TR/TE [ms]	Slice thick. [mm]	FOV [mm^2/3^]; Matrix size; In-plane resolution [mm^2^]	FA [°]	Additional Information
Diffusion / ADC mapping	3400/20	0.7	12x15; 96x128; 0.125x0.117	90/ 180	b value = 1500s/mm2 applied along 30 directions using 4 segments interleaved Spin Echo EPI with partial fourier factor = 1.5
T2 relaxometry	3100/ 8:8:136	0.7	20x20; 172x172; 0.116x0.116	90/ 180	Multislice Multiple Spin Echo sequence; TE increments of 8 ms from 8–136 ms;
T2 weighted	2000/22	0.7	20x20; 256x256; 0.078x0.078	90/ 180	Multislice RARE RARE factor 8
2D T1 weighted pre/post contrast agent injection	1000/0.6	1.0	15x15; 128x128; 0.117x0.117	90/ 180	Multislice RARE RARE factor 4
3D T1 weighted pre/post contrast agent injection	5/1.9	0.156	20x18.7x18.7; 128x120x120; 0.156x0.156	8.5	RF-spoiled FLASH Global RF excitation
T2* weighted	50/18	0.080	32x15x8; 400x188x100 0.080x0.080	12	Flow Compensated FLASH
Time of flight angiography	16/3.5	0.070	20x20x10 192x192x142 0.104x0.104	15	3D FLASH

### Image analysis

Image processing was undertaken in Amira 5.4 (FEI, Visualization Sciences Group). Due to morphological distortion caused by brain swelling and enlargement of the ventricles automatic image registration between time points was not performed in order to avoid incorrect local registration. Blood-brain barrier permeability (BBBD) was assessed by contrast-enhanced T1-w imaging. 3D non-enhanced T1w images were subtracted from enhanced T1w images; the difference images were evaluated for pathological enhancement by visual inspection. In pre- and post-contrast 3D T1w images, Gibbs ringing was suppressed and signal-to-noise-ratio enhanced using a 3D spatial Gaussian low-pass filter with a resulting effective isotropic resolution of 280μm. In case of significant motion between the pre- and post contrast image, images were motion corrected using a custom-made MATLAB code shifting the post contrast image iteratively by a fix amount of voxels to match the pre contrast image. Edema was determined by T2*, quantitative T2 and ADC values. For quantification of the T2 relaxation time multi-slice multi-spin-echo data were fitted after phase correction on a voxel-by-voxel basis with the monoexponential function A·e ^-(TE/T2)^ using a nonlinear least-squares fit procedure (MATLAB Release 2012b, The MathWorks, Inc., Natick, Massachusetts, United States). ADC values were calculated with the postprocessing algorithm provided by Paravision 6.0 (Bruker Bruker Biospin GbmH, Ettlingen, Germany). For analysis of vessel volume on T2*w images the same filter as in 3D T1w images was used with an effective isotropic resolution of 160μm.

Different regions-of-interest (ROI) were placed after anatomical delineation manually into the following structures on T2 and ADC maps: 1) olfactory bulb (OB)+rostral-migratory-stream (RMS), 2) dorsal-migratory-stream (DMS), 3) external capsule (EC), 4) cortex, 5) basal ganglia, 6) thalamus and 7) brainstem (BS) according to the Allen Brain Atlas [66]. RMS and DMS are only visible during disease, as in healthy mice they display the same signal intensity as the surrounding tissue. Therefore, ROIs were drawn at the estimated location of the structures on the scans without signal alterations in these areas. Microvascular pathology consisted of microhemorrhages and signs of microvascular sequestration determined by pathologic increase of vessel susceptibility contrast compared to baseline on T2*w images and vessel rarification on time-of-flight-angiography (ToF). Microhemorrhage volume was manually segmented on original T2*w datasets and vessel volume on filtered T2* datasets. Angiograms of ToF images were generated with maximum intensity projections and graded by degree of vessel rarification: 2 = normal/healthy vessel density, 1 = rarification of peripheral branches, 0 = decreasing vessel lumen of main branches and no visible peripheral branches.

### Histology

After transcardial perfusion with PBS, brains were removed and fixed in 4% PFA for 24h. Brains of severe (n = 6) and intermediate disease (n = 5) were cut sagittally brain on a vibratome (50μm floating sections) and processed for immunofluorescent staining. Brains of 4 severely sick ECM mice were cut sagittally at the level of the RMS on a microtome (1μm paraffin-embedded sections), embedded in paraffin and stained with conventional H.E. staining. Floating tissue sections were processed for co-immunofluorescent staining of doublecortin and Iba-1. Sections were first permeabilized with 1% triton in PBS for 20min, followed by a 30min incubation step in blocking buffer consisting of 10% goat serum. Subsequently, sections were incubated overnight at 4°C with primary antibodies against doublecortin (host species: goat; 1:500; Santa Cruz) and Iba-1 (host species: rabbit; 1:500; Wako Chemicals) followed by incubation with compatible Alexa 647 and Cy3-conjugated secondary antibodies (host species: donkey; 1:1000; Invitrogen/Jackson Immunoresearch) for 1 hour and 15 minutes. Stained sections were mounted on glass slides and then embedded in Mowiol mounting medium. Fluorescent staining was recorded using a confocal microscope LMS510 (Zeiss). For quantitative automated analyses of histological images, maximum intensity projections of five images (each with an optical section sickness of 1μm) were created to form one image stack. Binary masks were then produced using ImageJ (version 1.49s) [67] and further processed using custom-made MATLAB scripts and integrated FracLab codes (MATLAB Release 2012b, The MathWorks, Inc., Natick, Massachusetts, United States). To assess changes in microglial morphology consistent with microglial activation the respective images were screened for changes in cell shape and geometry using fully automated and operator independent lacunarity analysis. For instance, an image of no translational or rotational invariance and no gaps possesses a lacunarity of zero, whereas deviations from this state give an increasing lacunarity measure. When λ(ε) signifies the lacunarity at boxsize ε, then the average lacunarity Λ, also termed “graphical lacunarity”, is defined as: $\Lambda =\;\frac{\text{d ln}\left(\lambda \left(\epsilon \right)+1\right)}{\text{d ln}\left(\epsilon \right)}$ [27]. Naturally, the slope of the function y(x) = ln(*λ*(*e* ^x^)+1) with *x* = ln(*ϵ*) is best taken from the part of the graph where it is most linear. In our case this was the case for boxsizes *ϵ* = 4,8,16,32,64 (S1 Fig). Graphical lacunarity accurately represents quantitative measures of morphologically different states of microglial activation and increases with increasing microglial activation [68]. In addition, the number of microglial cells per mm^2^ was counted. Furthermore, morphological changes of neuroblast shape and geometry were evaluated by the index of linearity calculated as the quotient of major axis length and minor axis length (S2 Fig). The physiological usual spatial pattern of neuroblasts under physiological conditions is characterized by a highly ordered arrangement in linear chains, which is reflected in a high index of linearity (in control animals the index of linearity typically approaches 4 indicating a high degree of linear alignment/arrangement).

### Statistical analysis

Numerical data are presented as mean values ± standard-errors (SEM) unless indicated otherwise. All statistical analyses were computed with the software package STATA version 12.1 (StataCorp LP, College Station, TX, USA). Statistical testing was performed with non-parametric t-tests and equality tests using Wilcoxon’s matched-pair test for paired comparisons (procedure *signrank*) and Wilcoxon’s rank sum test for unmatched comparisons (procedure *ranksum*). P values < 0.05 were considered significant (two-sided).

## Supporting Information

S1 Fig Automated and quantitative lacunarity analysis of microglial activation. A, Representative Iba-1 image stacks (1024x1024 pixels in-plane, five 1μm thick images stacked by maximum-intensity-projection) at the level of the brainstem from a control (left), an animal with moderate disease (RMCBS 14, middle) and a severely sick animal (RMCBS 3, right) are displayed. Graphical lacunarity was assessed by averaging the values received by automated successive screening of the entire image area with different sized boxes (white squares with side lengths of 4, 8, 16, 32, 64 pixels). It is a measure for translational or rotational invariance and for the gappiness of an image. A value of 0 indicates no translational or rotational invariance and no gaps. B, Graphical lacunarity increases visibly during the activation process of microglial cells. The transformations in shape and geometry observed here for microglial cells are consistent with specific inflammatory activation and a highly regulated process of microglial activation (adapted from [68]). Scale bars indicate 50μm.
(TIF)

S2 Fig Automated and quantitative assessment of neuroblast migration. Representative source images of neuroblast alignment within the olfactory bulb in an uninfected control (first row, left) and an ECM mouse (second row, left) are displayed. To assess linearity and orientation of neuroblasts quantitatively and in an automated fashion binary masks were created (images on the right of source images): objects with less than 10 pixels in size were considered as noise (mean object size ~500 pixel). The index of linearity was calculated as the length ratio (a/b) between the length of the short axis (a) and long axis (b). In the olfactory bulb indices in controls reached values from 3.3 to 3.6 representing high linearity whereas values in all ECM mice showed a lower degree of linearity (indices from 2.2 to 2.8). A high degree of linearity indicates normal pattern of neuroblast chain migration. In ECM the spatial pattern of neuroblast arrangement is significantly altered (lower row) in comparison to that of controls (upper row), which is reflected in quantitatively lower indices of linearity.
(TIF)

S3 Fig Histological proof of microhemorrhages. A sagittal 1 μm HE section of one representative ECM mouse is displayed. Squares 1–4 are magnified on the right. Boxes 1 and 2 show microhemorrhages in the olfactory bulb, boxes 3 and 4 in the brain parenchyma in close vicinity to the RMS. Please note the extravasation of erythrocytes into the perivascular space (square 3 and 4).
(TIF)
