# Supplementary figures and images for: Experimental Cerebral Malaria Spreads along the Rostral Migratory Stream
Source: PLoS Pathog. 2016 Mar 10;12(3):e1005470. doi: 10.1371/journal.ppat.1005470 (PMC4786214; doi:10.1371/journal.ppat.1005470)

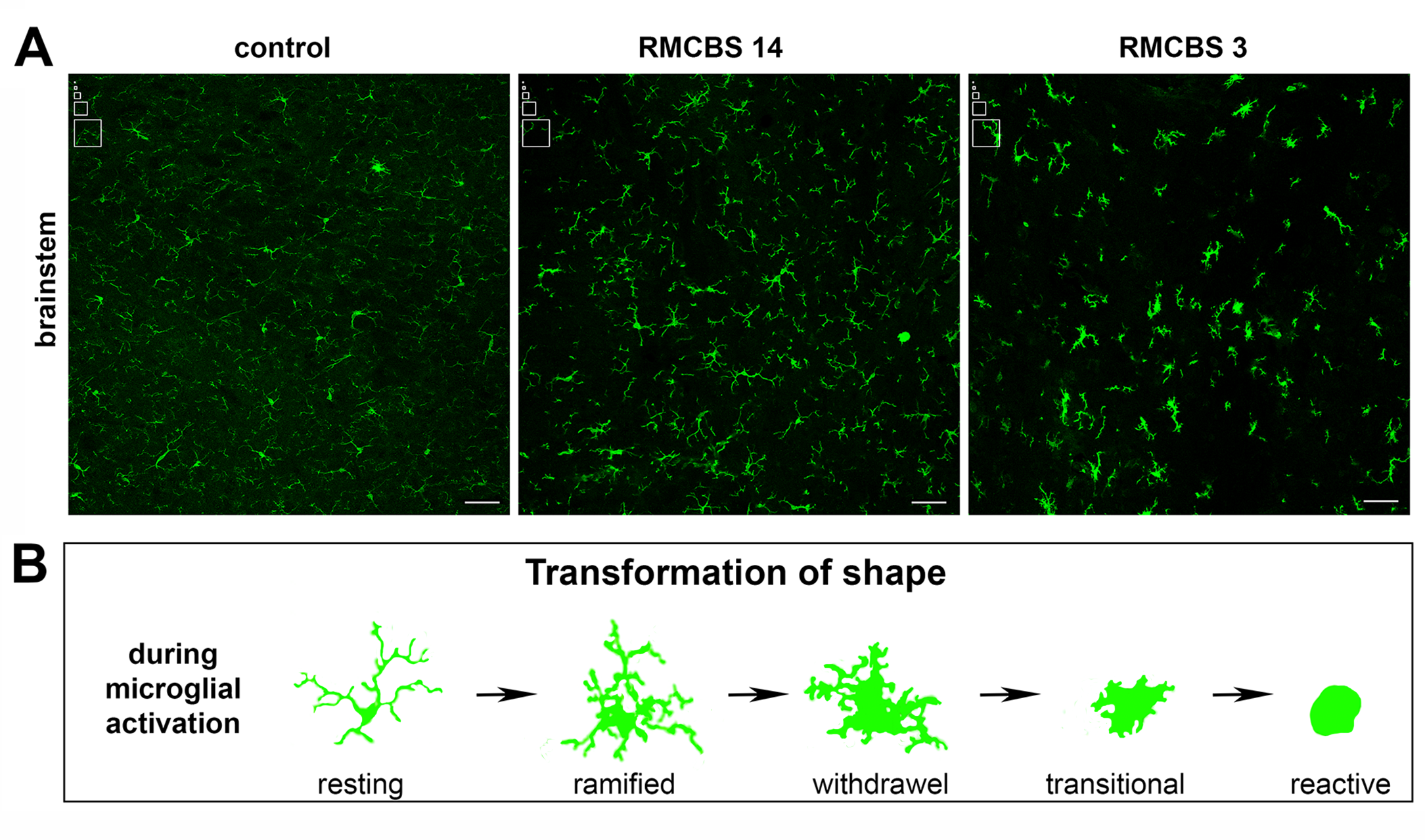

Supplement: S1 Fig — A, Representative Iba-1 image stacks (1024x1024 pixels in-plane, five 1μm thick images stacked by maximum-intensity-projection) at the level of the brainstem from a control (left), an animal with moderate disease (RMCBS 14, middle) and a severely sick animal (RMCBS 3, right) are displayed. Graphical lacunarity was assessed by averaging the values received by automated successive screening of the entire image area with different sized boxes (white squares with side lengths of 4, 8, 16, 32, 64 pixels). It is a measure for translational or rotational invariance and for the gappiness of an image. A value of 0 indicates no translational or rotational invariance and no gaps. B, Graphical lacunarity increases visibly during the activation process of microglial cells. The transformations in shape and geometry observed here for microglial cells are consistent with specific inflammatory activation and a highly regulated process of microglial activation (adapted from [68]). Scale bars indicate 50μm. (TIF) [file ppat.1005470.s001.tif]

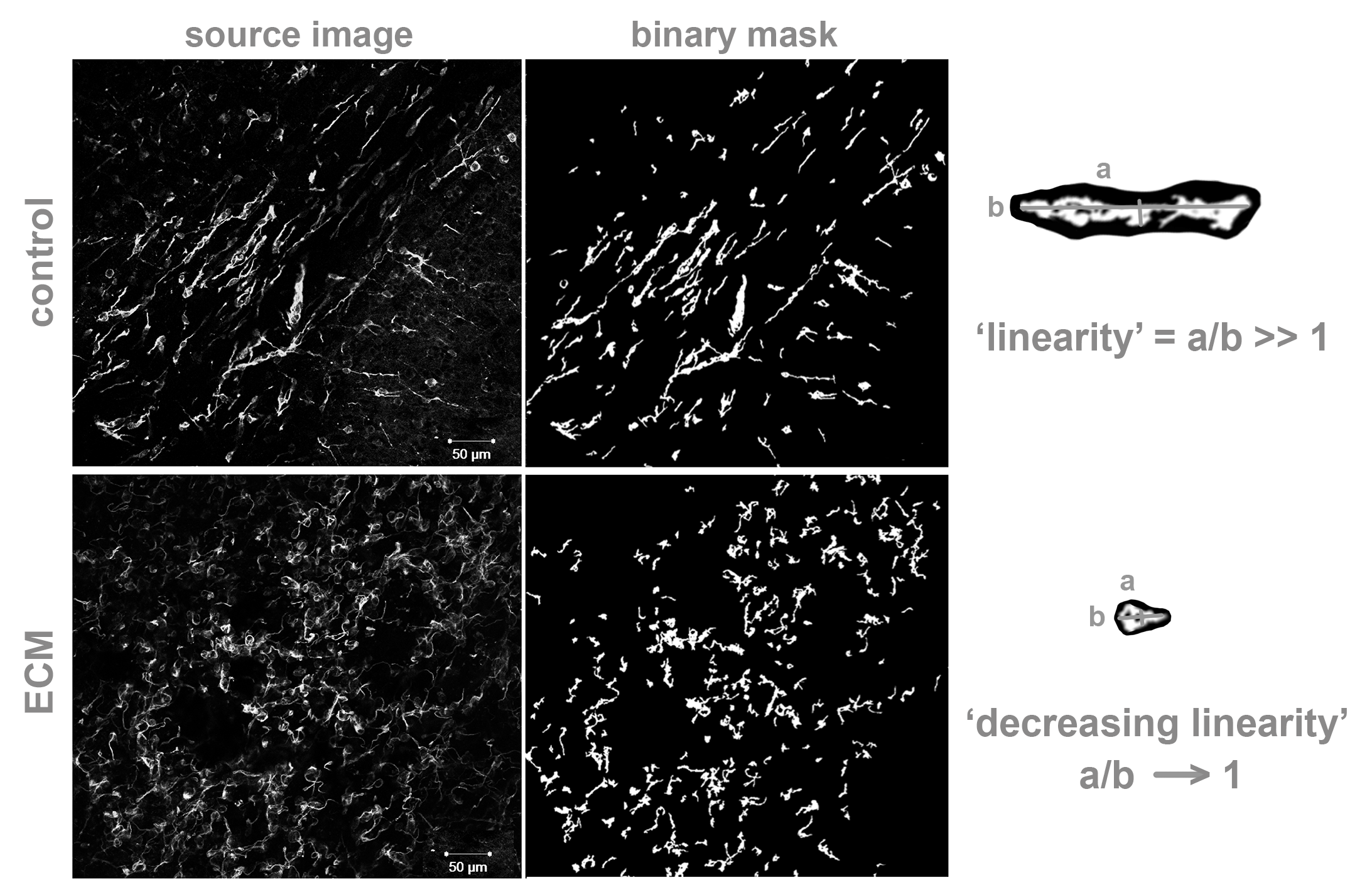

Supplement: S2 Fig — Representative source images of neuroblast alignment within the olfactory bulb in an uninfected control (first row, left) and an ECM mouse (second row, left) are displayed. To assess linearity and orientation of neuroblasts quantitatively and in an automated fashion binary masks were created (images on the right of source images): objects with less than 10 pixels in size were considered as noise (mean object size ~500 pixel). The index of linearity was calculated as the length ratio (a/b) between the length of the short axis (a) and long axis (b). In the olfactory bulb indices in controls reached values from 3.3 to 3.6 representing high linearity whereas values in all ECM mice showed a lower degree of linearity (indices from 2.2 to 2.8). A high degree of linearity indicates normal pattern of neuroblast chain migration. In ECM the spatial pattern of neuroblast arrangement is significantly altered (lower row) in comparison to that of controls (upper row), which is reflected in quantitatively lower indices of linearity. (TIF) [file ppat.1005470.s002.tif]

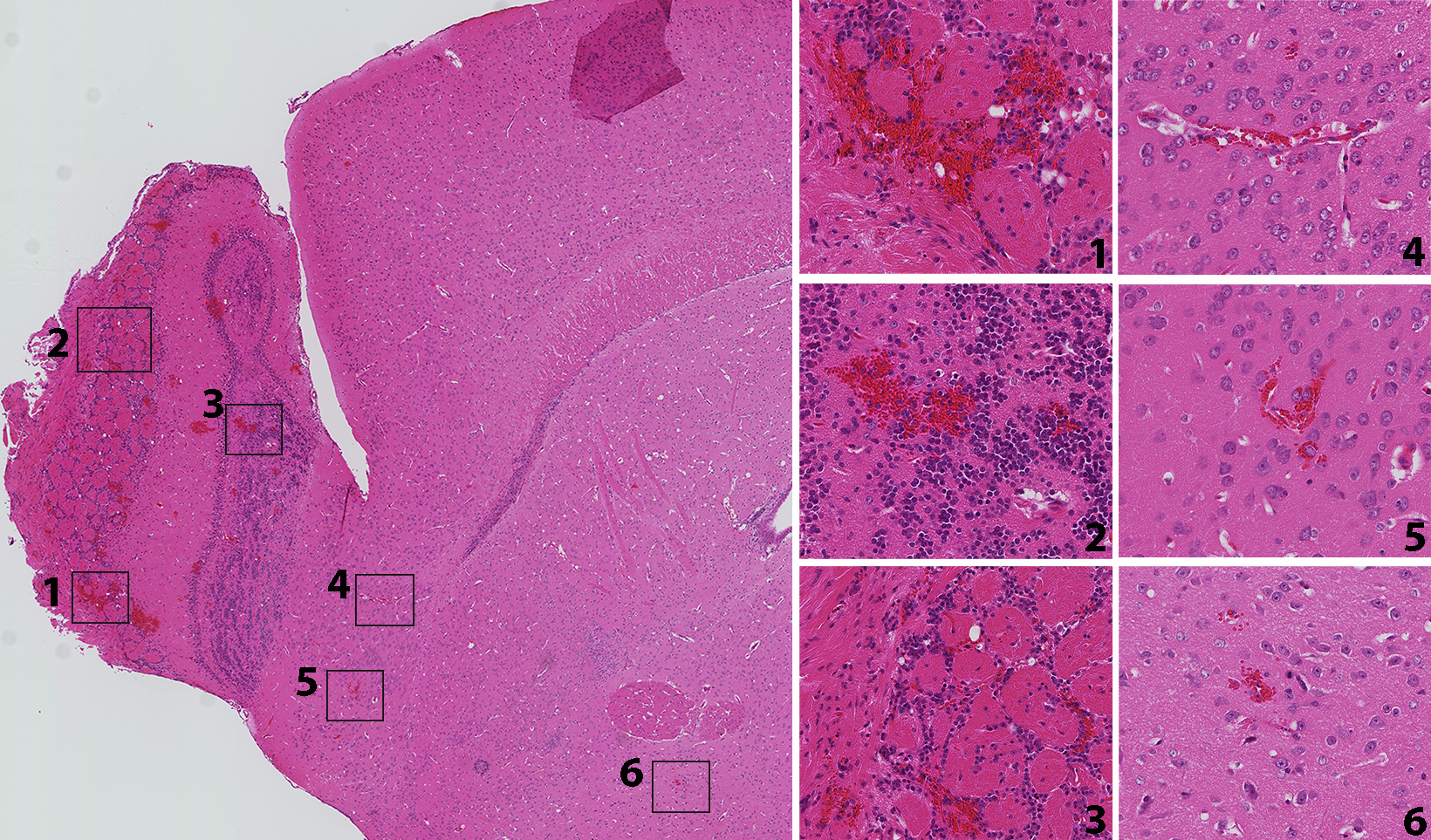

Supplement: S3 Fig — A sagittal 1 μm HE section of one representative ECM mouse is displayed. Squares 1–4 are magnified on the right. Boxes 1 and 2 show microhemorrhages in the olfactory bulb, boxes 3 and 4 in the brain parenchyma in close vicinity to the RMS. Please note the extravasation of erythrocytes into the perivascular space (square 3 and 4). (TIF) [file ppat.1005470.s003.tif]
